# A novel perspective suggesting high sustained energy expenditure may be net protective against cancer

**DOI:** 10.1093/emph/eoac012

**Published:** 2022-04-13

**Authors:** Peter A Biro, Frédéric Thomas, Beata Ujvari, Christa Beckmann

**Affiliations:** 1 Centre for Integrative Ecology, School of Life and Environmental Science, Deakin University, Geelong 3216, Australia; 2 CREEC, UMR IRD/CNRS/UM 5290, 911 Avenue Agropolis, BP 64501, 34394 Montpellier Cedex 5, France; 3 School of Science, Western Sydney University, Locked Bag 1797, Penrith, NSW 2751, Australia; 4 Hawkesbury Institute for the Environment, Western Sydney University, Locked Bag 1797, Penrith, NSW 2751, Australia

**Keywords:** metabolism, sustained energy expenditure, cancer, energetics

## Abstract

Energy expenditure (EE) is generally viewed as tumorigenic, due to production of reactive oxygen species (ROS) that can damage cells and DNA. On this basis, individuals within a species that sustain high EE should be more likely to develop cancer. Here, we argue the opposite, that high EE may be net protective effect against cancer, despite high ROS production. This is possible because individuals that sustain high EE have a greater energetic capacity (=greater energy acquisition, expenditure and ability to up-regulate output), and can therefore allocate energy to multiple cancer-fighting mechanisms with minimal energetic trade-offs. Our review finds that individuals sustaining high EE have greater antioxidant production, lower oxidative stress, greater immune function and lower cancer incidence. Our hypothesis and literature review suggest that EE may indeed be net protective against cancer, and that individual variation in energetic capacity may be a key mechanism to understand the highly individual nature of cancer risk in contemporary human populations and laboratory animals.

**Lay summary** The process of expending energy generates reactive oxygen species that can lead to oxidative stress, cell and DNA damage, and the accumulation of this damage is thought to be a major contributor to many ageing related diseases that include cancer. Here, we challenge this view, proposing how and why high energy expenditure (EE) may actually be net protective against cancer, and provide literature support for our hypothesis. We find individuals with high sustained EE have greater energetic capacity and thus can invest more in repair to counter oxidative stress, and more in immune function, both of which reduce cancer risk. Our hypothesis provides a novel mechanism to understand the highly individual nature of cancer, why taller individuals are more at risk, why physically active individuals have lower cancer risk, and why regular exercise can reduce cancer risk.

## INTRODUCTION

### Cellular damage, mutations and cancer

A prevalent theory for carcinogenesis is the mutation-centric theory stating that somatic nuclear mutations acquired over time lead to neoplasia, which then break free of the immune system and ultimately lead to cancerous transition [1, 2]. With the exception of congenital cancers, cellular damage and mutations leading to cancer can arise from external risk factors (UV, toxins), infections and inflammation. However, they also arise from internal risk factors associated with normal energy production and cell division that generate toxic and mutagenic reactive oxygen species (ROS), leading to an increase in cancer probability with chronological and physiological age (reviewed by References [3, 4]).

### Cancer as a consequence of energy expenditure over time?

A current paradigm is that energy expenditure (EE) is tumorigenic, due to the continual production of ROS and other toxic by-products of EE, leading to oxidative stress, damage to cells and DNA, and ultimately cancer (reviewed by References [3, 5–9]). These papers address and review literature emphasizing the role of ROS in cancer incidence, highlighting that high mass-corrected metabolic rate (indeed a direct measure of EE) is expected to lead to higher cancer risk, providing many literature citations supporting this view. Of course, antioxidants and other mechanisms exist that can mitigate the effects of ROS production, but these are imperfect and often insufficient; this leads to a logical assumption that these negative effects are repeated over time and may accumulate, ultimately leading to cancer. Consequently, this paradigm is at the heart of explaining why age is the strongest predictor of cancer risk [3, 9, 10] and why physiological ageing might be an even stronger predictor [4].

### Reasons why cancer may not be substantially related to EE

Although the arguments linking cancer to EE are logical and suggest that EE is at least partly tumorigenic, this reasoning assumes antioxidants and other mechanisms are insufficient to mitigate the effects of ROS production. This assumption leads naturally to at least two predictions at the within-species level that are either false, or likely to be false. First, such arguments suggest that taller individuals in humans should have lower cancer risk due to their necessarily lower resting EE and correspondingly lower ROS production rate [11, 12], but we actually observe the opposite; taller humans are well-known to have a higher risk of cancer, across a wide range of different cancers [13, and review therein]. This trend also exists in other animals, such as dogs [14], but these trends are not easily separated from effects of an increased number of cells and greater early-life growth, which may also play a role. Second, individuals that expend more energy through regular exercise and post-exercise recovery produce more ROS that should increase cancer risk, yet physical activity is well-known to lower cancer risk across a range of different cancers [15] see also References [16–24]. Clearly, these observations are opposite to the predictions of the paradigm that proposes links between EE, oxidative stress and cancer, indicating that there is something at least partially incorrect with the view that high EE is tumorigenic. So how can we reconcile this?

## HOW AND WHY HIGH EE CAN BE NET PROTECTIVE AGAINST CANCER

Opposite to current viewpoints, we argue here that high EE may have a net protective effect against cancer, despite high ROS production. In our review below, we focus on individual variation in energetics within species and show that individuals differ in levels of resting, maximum and sustained daily EE. In turn, individuals exhibiting consistently higher EE are known to have greater capacity to acquire, ingest and process food into energy, which is what we refer to here as ‘energetic capacity’ (see Box 1 for a full explanation and literature support).

Box 1. How and why individuals can vary in energetic capacity

An individuals’ energetic capacity can be defined as the ability to sustain high total EE, and to quickly up-regulate EE when required [67]. Energetic capacity can be approximated by commonly used whole-organism aerobic metabolism measures (=EE), such as resting and maximum metabolic rate (RMR, MMR) and VO_2max_ (which is equivalent to MMR). These three measures of aerobic metabolism are positively correlated with measures of sustained EE at the among-and within-individual level, which include total daily energy expenditure (DEE or TEE), and the capacity to up-regulate energy output when demands increase (reviewed and discussed by References [67–73]).

Repeatability and heritability of RMR, MMR, VO_2max_, and sustained EE (DEE), when measured under conditions of abundant food, further demonstrate innate (intrinsic) variation among individuals in their capacity to process food into useable energy [74–77]. This indicates individuals vary in their capacity to acquire and generate energy, and this intrinsic energy limitation is further demonstrated by numerous artificial selection studies using rodents, whereby upward selection on either metabolism or on physical activity results in correlated increases in RMR, MMR, food intake, aerobic capacity (VO_2max_), spontaneous activity in home cages, running wheel duration and speed, and even reproductive output (reviewed by References [67, 77]). Together, these studies provide compelling evidence that when food is abundant, as is the case for contemporary humans, then energy acquisition (intake rate), energetic output and costly energetic processes and activities can all be positively correlated with each other. Indeed, an absence of trade-offs is a theoretical expectation when variation among individuals in energy acquisition is large [25, 26].

Observations such as those described above support the ‘performance’ model of energy management, whereby higher resting EE (RMR, i.e. maintenance energy costs) reflects the energetic cost of larger and more active organs involved in energy processing that are required to support higher energy output on a sustained daily basis [71, 77, 78]. Indeed, many of the selection studies on rodents described above, and other studies, also show that individuals with higher EE also possess larger internal organs, such as intestines, liver, kidneys and heart that have high mass-specific metabolism and comprise the majority of resting EE (reviewed by References [67, 71, 77]).

All together, these observations and viewpoints share some similarity to the hypothesis that humans have a constrained TEE [79]. Indeed, this has been shown in other mammals as we discuss above, highlighting intrinsic energy acquisition limits. However, where our ideas differ from that of Pontzer (2018) is in our focus on between individual variation in capacity, emphasizing that each individual has a maximum and thus constrained TEE, whereby individuals with very high TEE (supported by high RMR) have larger energy capacity and thus can allocate energy to multiple competing demands with little or no evidence of trade-offs.

Greater energetic capacity permits abundant energy to allocate towards multiple demands simultaneously. This can occur because humans, and animals fed ad lib in captivity, are rarely energy limited by the environment, but rather are internally limited by their bodies’ ability to process food into useable energy. Thus, high-capacity individuals may generate the energy required to not only counteract ROS via costly antioxidant production, but also to fight any neoplasia that emerge via adaptive and innate immune function to stop the progression of neoplasia into cancer, without necessarily showing evidence of energetic trade-offs with other bodily functions [25, 26]. Thus, if we look solely at the negative effect of EE on ROS production, we see only part of the story and likely draw the wrong conclusion regarding its contribution to cancer risk.

**Figure 1.**
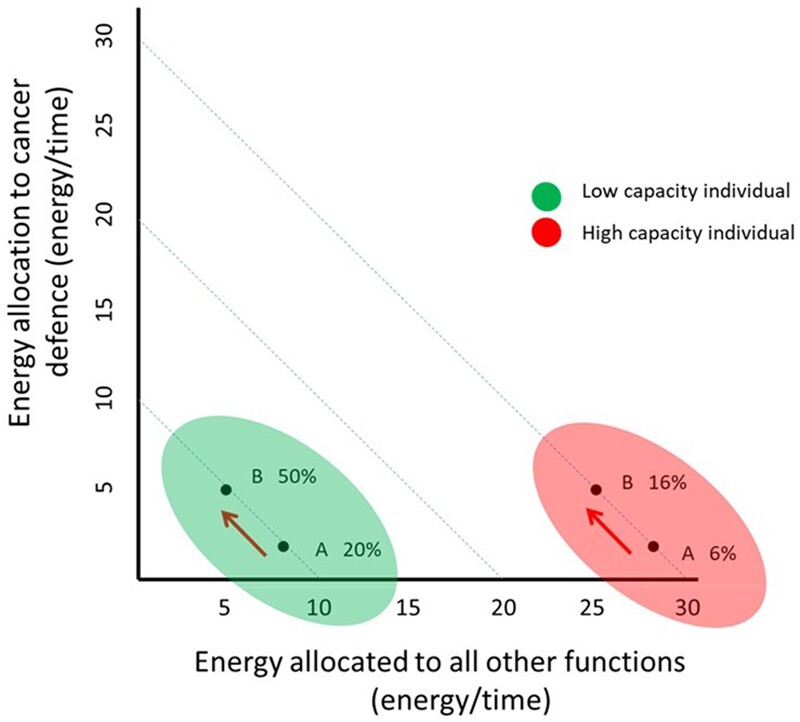
Schematic diagram of total energy available to the body (=energetic capacity, see Box 1) and its allocation to anti-cancer processes (antioxidant, repair and immune function) vs all other energetic demands. Two individuals of equal mass are depicted, but where one has low capacity (shaded ellipse on left, in green) and the other has high capacity (ellipse on right, in red), with 10 (low capacity individual) and 30 (high-capacity individual) arbitrary energy units to spend, per unit time. Lower point for each individual (**A**) indicates the proportion of the total energy budget represented by cancer defence costs of two units; arrows illustrate the change in within-individual allocation when cancer defence costs increase from two to five units (from A to **B**). This illustrates how this increase represents a major proportion of the energy budget for a low capacity individual (from 20% to 50%), and thus a large within-individual trade-off; by contrast, this increase in a high-capacity individual is a small relative increase (from 6% to 16%) with a small within-individual trade-off. Importantly, at the among-individual level however there is no trade-off (both individuals increase defence costs by three units)

Our viewpoint does not imply that trade-offs do not exist—rather, trade-offs can be absent when looking at the among-individual level, but substantial within-individual trade-offs may exist for individuals with low energetic capacity. This may occur when a given energetic challenge like fighting neoplasia emerges, that may represent a large proportion of the energy budget of a low capacity individual, whereas this same challenge may represent a small proportion of a high-capacity individuals’ energy budget (illustrated and further explained in Fig. 1).

In the material that follows, we present arguments and review literature that together provide compelling (but preliminary) support for our hypothesis that higher EE may be net protective against cancer due to greater associated energetic capacity, at least for laboratory animals fed ad lib and humans in much of the world in recent decades. Throughout, our emphasis is on inter-individual differences in EE, helping to answer the call to identify mechanisms that might account for individual variation in susceptibility to cancer [27] and to better understand the role of individual variation in metabolism in relation to health and disease risk [28].

## LITERATURE SUPPORT FOR HYPOTHESIS

### High EE does not necessarily equate to more oxidative damage

EE results in the production of ROS, which is known to be an important factor underlying cellular and whole-organism senescence, and a factor increasing cancer risk [29]. However, ROS can only exert negative effects on cells and increase mutation risk if ROS production rate exceeds the capacity of an individuals’ antioxidant defence and repair mechanisms [29]. There is a long tradition of assuming that high rates of EE necessarily force trade-offs, where individuals with high EE cannot also fuel increased antioxidant function [30]. However, it appears that oxidative damage does not necessarily tend to increase with increased EE, because when food resources are not limited, individuals with high EE can defend against the effects of high ROS (reviewed and discussed by Reference [30]).

There is now substantial evidence that individuals with consistently high EE can alleviate effects of toxic by-products via antioxidants. For example, rats artificially selected for high maximum aerobic metabolic rate (=VO_2max_) also have higher resting EE, are more active and run greater distances on a daily basis, and yet they show lower oxidative stress than down-selected rats with low EE [31]. Similarly, mice artificially selected for high resting EE were also more active on a sustained basis, meaning higher EE, but again they too show lower oxidative stress than down-selected mice with low EE [32]. These artificial selection studies highlight the antioxidant ability of individuals with high innate EE, demonstrating a heritable component.

Experimental manipulation of activity levels in humans and rodents further demonstrate how individuals with high EE can defend against the effects of the increased ROS. For example, mice permitted to do voluntary exercise on running wheels throughout life had lower oxidative stress than mice without running wheels [33], as did rats in a similar study [34]. Furthermore, humans that have higher EE due to exercise have greater antioxidant capacity and correspondingly lower oxidative stress than sedentary individuals [35, 36]. Similarly, higher aerobic capacity (VO_2max_, a reliable predictor of physical activity) is associated with lower systemic oxidative stress in humans [37] and in rats [38].

There is also now some limited evidence suggesting that the widespread assumption that ROS are produced in direct proportion to increases in EE may not always operate as expected. For example, in a study using fish, individuals with high resting metabolic rate (RMR) had lower ROS (H_2_0_2_) than individuals with low RMR [39]. Indeed, while oxidative stress is thought to be at heart of senescence and reduced longevity, as well as cancer risk, a few compelling studies contradict this view. For example, isolines of fruit flies (*Drosophila* sp.) with high EE showed no signs of greater senescence or reduced longevity compared to isolines with low EE [40]. Similarly, rats selected for high VO_2max_ and which display high physical activity and high EE throughout life senesce slowly, and live longer than rats selected for low EE [41]. In addition, these up-selected rats with high EE are also less susceptible to cancer [42], and have an enhanced ability to repair oxidative cell damage through autophagy [43]. This result was also mirrored in a similar artificial selection study using mice [44]. Further, insect genotypes with high intrinsic growth rates (and correspondingly high EE), and showing higher capacity for rapid growth following a period of food shortage, live longer and had more antioxidant enzymes, and tended to experience lower oxidative damage than genotypes with slow intrinsic growth rates [45].

In humans, a long-term prospective study of twins demonstrated that greater longevity is associated with lower cancer risk [46], a result that is similar to the study using artificially selected rats (above). And finally, a large meta-analysis indicates that elite athletes with their high EE and high energetic capacity enjoy greater longevity and lower cancer incidence than the general population [47]. Together, these studies are just a few of the many examples which highlight that oxidative stress is not a necessary outcome of high EE and production of new biomass, nor reduce longevity, as is usually assumed (reviewed by References [30, 48]).

### High EE can enhance immune function to fight emerging neoplasia

Immune function plays a critical role in cancer development, with both the innate and the adaptive immune system preventing neoplasia from transforming into invasive cancer (reviewed in References [10, 49, 50]). Indeed, cancer resistance can evolve through evolution of enhanced immune function [51].

Immune function is energetically costly, and not surprisingly, increases in EE are commonly associated with individuals fighting cancer that can challenge ones energetic capacity [52, 53]. Individuals with high EE may be able to sustain the costs of effective adaptive and innate immune function due to preferential allocation of energy towards immunity. Alternatively, individuals with high EE and high energetic capacity may be able to fuel effective immune function without trade-offs, as we review next.

Artificial selection experiments using rodents provide some of the most compelling evidence that individuals with high EE also sustain high immune function. For example, mice selected for high resting EE had enhanced immune function and larger lymph node mass and spleen than mice selected for low resting EE, indicating that elevated energy capacity (reflected by high resting EE, see Box 1) permits greater immune response, while also permitting greater sustained activity rates [54]. In another example, rats selected for low and for high EE were exposed to breast cancer cells, and while high EE rats effectively resisted the spread of cancer, low-EE rats did not and, additionally, lost weight over time [42]. The latter result in particular, highlights that the energy budget of low-EE rats was challenged resulting in weight loss, in contrast to high-EE rats that appear to have spare energetic capacity for rapid immune response.

There is also evidence to suggest up-regulation of EE via regular exercise (a sustained increase in EE) increases energetic capacity and permits greater immune response to fight cancer. Rapid and lasting increases in resting and maximum EE (RMR and VO_2max_) in response to ongoing exercise regimes are often associated with subsequent greater immune function in humans and rodents (reviewed in References [55–60]). For example, just 4 weeks of exercise prior to cancer induction in mice elevated NK cell activity, lowered cancer incidence and reduced tumour growth [61]. Similarly, 9 weeks of training greatly increased immune function in another study in mice [62], and sustained exercise in mice reduced oxidative stress and inflammation levels [33].

## SUMMARY AND IMPLICATIONS OF OUR HYPOTHESIS

Our review and discussion suggest that there is already some substantial evidence against the hypothesis that high EE necessarily leads to oxidative stress, indicating high EE may not be tumorigenic as widely assumed. There is also already some substantial evidence that high EE may in fact be net protective against cancer due to greater antioxidant and immune investment by individuals that possess high energetic capacity.

In the first instance, if high EE is indeed net protective against cancer, this provides a viable mechanism to help us understand the highly individual nature of cancer risk. Individuals differ dramatically in terms of cancer risk even when ‘all else is equal’ (risk factors, sex, age etc. [63]), and our review suggests that energetic capacity, which differs among individuals may help explain the individuality of cancer risk. Recently, an evaluation of several hypotheses for cancer development revealed that context-dependent selection was necessary and sufficient to explain patterns of cancer incidence, and that individual variation in mechanisms that affect ‘tissue microenvironment’ is the most promising of research directions [27]. Here, we suggest whole-organism energetic capacity may just be such a mechanism. This mechanism could also explain individual differences in ‘cancer-immune’ set points, which refer to immune-responsiveness that differs among individuals to affect cancer incidence [56, 60, 64].

Secondly, our review indicates that researchers might reconsider the view that EE is necessarily tumorigenic. In particular, we highlight that the traditional view that high EE necessarily forces substantial trade-offs with other important functions is not necessarily true, that oxidative stress is not necessarily a cost of high EE, and that longevity more generally need not be sacrificed at the expense of high EE. While metabolic explanations for Peto’s paradox may still apply across species, it clearly cannot explain the higher cancer risk in taller humans and larger dog breeds that necessarily have lower RMR (discussed above).

Further research to investigate antioxidant, cell repair capacity and immune function and cancer incidence in relation to energetic capacity and EE are required to further validate the patterns documented here. This would require repeated measures of RMR, daily EE, ROS production and antioxidant production using a multivariate repeated measures approach, embedded within a long-term study of cancer incidence of individual humans or other animals; clearly, a difficult and time-consuming approach, but one that is required given that measures of metabolism are labile traits. Use of models, particularly mice and rats within one of the several ongoing artificial selection studies might be the most productive and informative as our review already indicates.

If our hypothesis has merit, it does imply an important role for early developmental interventions to encourage physical activity and sustaining activity throughout life to encourage and maintain high energetic capacity, not just for cancer prevention, but also health more generally. This view is supported by large-scale studies indicating that high EE improves health and reduces mortality of all types. Indeed, aerobic capacity (=VO_2max_, a measure of physical activity and energetic capacity) tends to be a stronger predictor of cardiovascular health and all-cause mortality (including cancer) than actual EE in physical activity [65, 66], suggesting energetic capacity as a key and direct mechanism.

In conclusion, this new hypothesis we raised here is one that seems to have sufficient support to justify pursuing this topic further, especially as it has important implications for how we view and study associations between energetics, effects of ROS, immune function, activity and cancer risk. We hope it encourages further studies of the sort we have identified in our review.

**Conflict of interest:** None declared.
